# Comparing ant morphology measurements from microscope and online AntWeb.org 2D z‐stacked images

**DOI:** 10.1002/ece3.9897

**Published:** 2023-03-19

**Authors:** Sándor Csősz, Ferenc Báthori, Zoltán Rádai, Gábor Herczeg, Brian L. Fisher

**Affiliations:** ^1^ ELKH‐ELTE‐MTM Integrative Ecology Research Group Budapest Hungary; ^2^ Department of Systematic Zoology and Ecology Institute of Biology, ELTE‐Eötvös Loránd University Budapest Hungary; ^3^ Lendület Seed Ecology Research Group Institute of Ecology and Botany, Centre for Ecological Research Vácrátót Hungary; ^4^ Entomology California Academy of Sciences San Francisco California USA

**Keywords:** AntWeb.org, morphometry, reproducibility, statistical agreement, systematic bias, virtual collection

## Abstract

Unprecedented technological advances in digitization and the steadily expanding open‐access digital repositories are yielding new opportunities to quickly and efficiently measure morphological traits without transportation and advanced/expensive microscope machinery. A prime example is the AntWeb.org database, which allows researchers from all over the world to study taxonomic, ecological, or evolutionary questions on the same ant specimens with ease. However, the reproducibility and reliability of morphometric data deduced from AntWeb compared to traditional microscope measurements has not yet been tested. Here, we compared 12 morphological traits of 46 *Temnothorax* ant specimens measured either directly by stereomicroscope on physical specimens or via the widely used open‐access software tpsDig utilizing AntWeb digital images. We employed a complex statistical framework to test several aspects of reproducibility and reliability between the methods. We estimated (i) the agreement between the measurement methods and (ii) the trait value dependence of the agreement, then (iii) compared the coefficients of variation produced by the different methods, and finally, (iv) tested for systematic bias between the methods in a mixed modeling‐based statistical framework. The stereomicroscope measurements were extremely precise. Our comparisons showed that agreement between the two methods was exceptionally high, without trait value dependence. Furthermore, the coefficients of variation did not differ between the methods. However, we found systematic bias in eight traits: apart from one trait where software measurements overestimated the microscopic measurements, the former underestimated the latter. Our results shed light on the fact that relying solely on the level of agreement between methods can be highly misleading. In our case, even though the software measurements predicted microscope measurements very well, replacing traditional microscope measurements with software measurements, and especially mixing data collected by the different methods, might result in erroneous conclusions. We provide guidance on the best way to utilize virtual specimens (2D z‐stacked images) as a source of morphometric data, emphasizing the method's limitations in certain fields and applications.

## Graphical abstract

Our findings shed light on the fact that a relatively high degree of uncertainty of software measurements of images deposited in virtual collections is expected. Therefore, morphometric data collected from virtual specimens must be treated with extreme care. In our paper, the method's limitations in specific fields and applications are discussed, and guidance on the most reliable utilizing morphometric data taken from virtual representatives is provided.

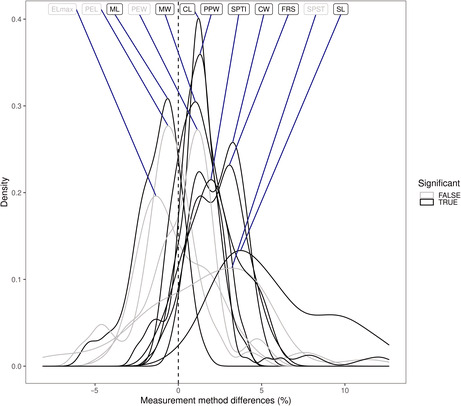

## 1 INTRODUCTION

Morphological data constitute fundamental information used within the life sciences, from descriptions of new species to studies of evolutionary questions (Cabral et al., 2012; Deans et al., 2015; Elgar et al., 2018; Thompson et al., 2011). Modern morphological examination of organisms, particularly in biodiversity research, often requires quantitative, morphometric‐based approaches (Lieber, 2021; Snodgrass, 2018). Indeed, morphometrics, one of the most widely used quantitative approaches to studying morphology, has long been a popular approach in taxonomy and systematics (e.g., Baur et al., 2014; Christodoulou et al., 2020; Johnson et al., 2018; Longino & Branstetter, 2020; Michaloudi et al., 2018; Wagner et al., 2017). It is also a favored source of data to pursue questions in morphological evolution (Dehon et al., 2014; Lawing & Polly, 2010; Wagner et al., 2018), and constitutes a sound methodology for detecting allometries in developmental biology (Chiu et al., 2015; Demuth et al., 2012; Laciny, 2021). Even in the era of rapidly advancing DNA sequencing technologies (Luo et al., 2018; Puillandre et al., 2012; Rannala & Yang, 2020), morphometry retains its prestige, as this approach is considered one of the most promising ways to find links between molecular conclusions and name‐bearing types, that is, zoological nomenclature (Alitto et al., 2019; Renner et al., 2018).

The classic stereomicroscopic measurement method has long been the standard approach for the morphological examination of specimens. However, image‐based morphological methods have become increasingly popular (Hoenle et al., 2020). Furthermore, unprecedented technological advances in digitization and steadily expanding open‐access databases with mass sources of phenotypic information [e.g., AntWeb (www.antweb.org); FaceBase (https://www.facebase.org/); MosquitoLab ‐ Wingbank (www.wingbank.butantan.gov.br)] yield new opportunities in science (Bellin et al., 2021; Hoenle et al., 2020; McQuin et al., 2018; Psenner, 2018; Samuels et al., 2020; Virginio et al., 2021; Wang et al., 2020). These methods have opened new ways for scientists to study virtual specimens (Hsiang et al., 2018), but their use usually requires high‐quality digital data sources (Davies et al., 2017; Lürig et al., 2021). Nevertheless, this possibility is relatively new to the biological community. Beyond the opportunity this technology brings, the knowledge acceleration generated by digitalization poses novel challenges. For instance, we need to learn more about the potential benefits and costs of these new digital measuring methods compared to traditional microscopic examination of specimens in entomology. As previous studies have shown, morphometric measurements are subject to some degree of error due to factors such as the experience of the researchers performing the measurements, the magnification of the equipment, and the size of the measured characters (Csősz et al., 2021; Takács et al., 2016; Yezerinac et al., 1992). Such problems can be easily overcome by using software to take measurements from high‐resolution digital images. However, different measurement methods may also yield discrepant results (Wylde & Bonduriansky, 2021), especially when examining minor characters prevalent in insects.

AntWeb launched in 2002 (Fisher, 2002), is the most comprehensive online means to access museum ant collections. AntWeb contains, as of October 2022, 791,974 specimen records, of which 56,224 specimens are imaged and contain a total of 244,438 images across 13,368 valid species and subspecies, 4911 morphotaxa, and further ca. 400 unrecognized and unidentifiable names. Since 2002, AntWeb has been regularly cited as a resource in ant research; a Google Scholar search of the term “Antweb” reveals over 3000 publications through September 2022. These publications include standard systematic research but also other fields. For example, Báthori et al. (2017) used this resource to screen images for fungal ectoparasites. Marques et al. (2018) developed a new method for identifying ant genera with a set of convolutional neural networks that contributed significantly to the extraction of taxonomic knowledge without human intervention. Idec et al. (2023) used Antweb images for the first global assessment of macroecological and macroevolutionary patterns of color in ants. Helms (2022) used AntWeb records to study large‐scale geographic variation in ant mating seasons. Klunk et al. (2022) used Antweb images to study melanism evolutions in worker ants. The images in the database have also been used several times to provide educational materials (MacGown & Whitehouse, 2009) and morphological measurements for various studies (Ferguson‐Gow et al., 2014; Leong et al., 2015). Despite AntWeb's booming popularity in various research fields, its reliability compared to traditional, direct microscopic measurements of specimens has never formally been tested.

In the present paper, we aimed to compare data gathered by software measurements made on digital images from the AntWeb repository to data gathered by the traditional microscopic measurement method on linear measurements of 12 traits using the same specimens (*N* = 46) from the *Temnothorax* ant genus. We note that moderate‐to‐high agreement (repeatability, reproducibility) between methods alone does not guarantee that separate analyses of datasets gathered by different methods applied on the same objects will yield the same patterns or mixing datasets. Despite high statistical agreement, there can be trait value dependence in the level of agreement; datasets can have different variances; and there can always be systematic bias between methods that does not affect the statistical agreement. Therefore, to capture as many sources of potential error as possible, we quantified (i) the agreement between the measurement methods (microscopic vs. software), (ii) the trait value dependence of the agreement, (iii) the coefficients of variation produced by the different methods, and (iv) the systematic bias between the methods in a mixed modeling‐based statistical framework.

## 2 MATERIALS AND METHODS

### 2.1 Data sources and sampling

**FIGURE 1:**
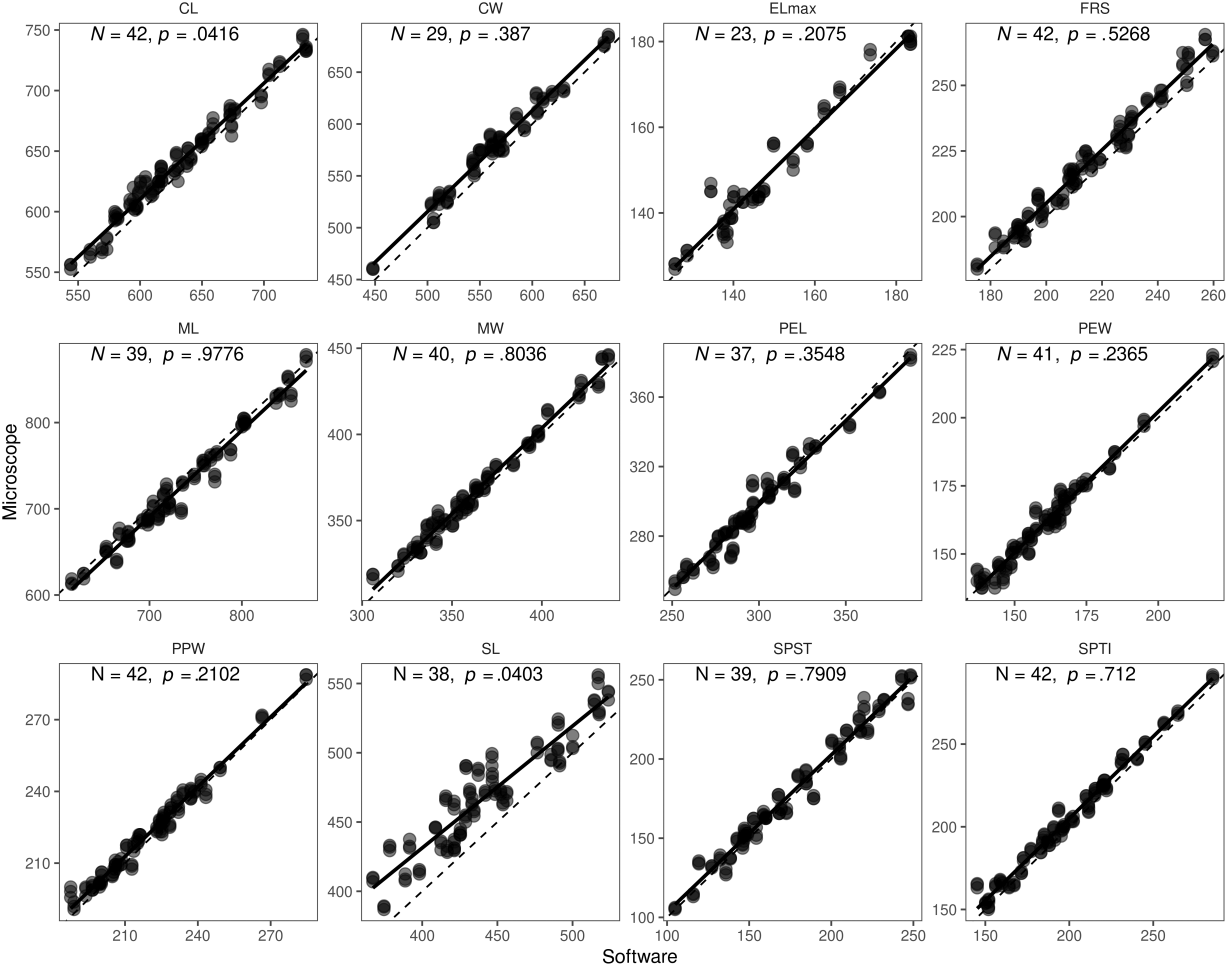
The association between microscope and software measurements was analyzed separately for the tested traits. *N* is the number of specimens for which both measurement methods could be used for the given trait. *p*‐values represent the significance of the intercept not being zero (i.e., the significance of average value differences between methods, see Section 2 for details). Dashed lines denote the expected association in the case of perfect agreement between methods; solid lines represent the regression slopes from the fitted models. Abbreviations in the figure are as follows: CL, cephalic length; CW, cephalic width; Elmax, diameter of the compound eye; FRS, frontal carina distance; ML, mesosoma length; MW, mesosoma width; PEL, petiole length; PEW, petiole width; PPW, postpetiole width; SL, scape length; SPST, propodeal spine length; SPTI, apical propodeal spine distance.

AntWeb is the world's largest online database of images, specimen records, and natural history information on ants (AntWeb, 2022). Based on current statistics (ver. 8.8.), 791,927 specimen records and 244,065 total specimen images contributed from all over the world can be found on AntWeb. At least three high‐quality photos of most individuals, taken from three perspectives (frontal, dorsal, and profile), are uploaded to illustrate critical taxonomic characters (Figure 1). Images were taken using a Leica DF425 camera using the same image format settings under a Leica LED5000 HDI Dome Illuminator and followed a standard protocol for AntWeb (https://www.antweb.org/web/homepage/Imaging_Manual_LAS38_v03.pdf). Images taken with telecentric lenses are not subject to perspective distortion due to changes in focal distance. However, lenses that are not telecentric are susceptible to distortions which must be corrected during the Z‐stacking process. The 2D z‐stacked images of the virtual specimens were created from a stack of images across the focal range using the focus‐stacking software in Leica Application Suite software (v3.8). We have randomly chosen 46 ant worker specimens belonging to 20 *Temnothorax* species from the Hymenoptera collection of the Hungarian Natural History Museum that were also included in AntWeb, with digital photography conducted in the standard way by Estella Ortega, Flavia Esteves and Michele Esposito. Only perfectly intact specimens with well‐aligned images were included in this study.

### 2.2 Morphometrics

All microscopic measurements were made with an ocular micrometer using an Olympus SZX 16 stereomicroscope equipped with an ocular micrometer at a magnification of 80× (for larger body parts) and 160× (for smaller traits) on physical specimens by FB. All microscopic measurements were made in μm using a pin‐holding stage, permitting rotations around the X, Y, and Z axes. Every measurement was repeated three times. Repeats were done in random order, on different days, and were entirely independent, that is, the full process from retrieving the individual from the collection to doing the actual measurements was repeated.

Software measurements were made with TpsDig ver 2.32 (Rohlf, 2001) software by FB. TpsDig is a Windows program designed to digitize landmarks and outlines for geometric morphometric analyses. Before starting the measurements, the software was calibrated to the scale in each image examined. We measured the same set of characters with both the software and the microscope. The complete list of measured characters defined by Csősz et al. (2015) is available in Table 1. All morphometric data are given in μm and provided in Table S1.

**TABLE 1:** Abbreviations of morphometric characters, definition of measurements.

Abbreviation	Definition
CL	Maximum cephalic length in the median line. The head must be carefully tilted to the position providing the actual maximum. Excavations of hind vertex and/or clypeus reduce CL.
CW	Maximum width of the head, including compound eyes.
ELmax	Maximum diameter of the compound eye.
FRS	Frontal carina distance. Distance of the frontal carinae immediately caudal of the posterior intersection points between frontal carinae and the torular lamellae. If these dorsal lamellae do not laterally surpass the frontal carinae, the deepest point of scape corner pits may be taken as the reference line. These pits take up the inner corner of the scape base when the scape is directed caudally and produces a dark triangular shadow in the lateral frontal lobes immediately posterior to the dorsal lamellae of the scape joint capsule.
ML	(Weber length) Mesosoma length from caudalmost point of propodeal lobe to transition point between anterior pronotal slope and anterior pronotal shield. Preferentially measured in lateral view; if the transition point is not well defined, use dorsal view and take the center of the dark‐shaded borderline between pronotal slope and pronotal shield as the anterior reference point.
MW	Mesosoma width. In workers, MW is defined as the longest width of the pronotum in the dorsal view, excluding the pronotal spines.
PEL	Diagonal petiolar length in lateral view; measured from anterior corner of subpetiolar process to dorso‐caudal corner of caudal cylinder.
PEW	Maximum width of petiole in dorsal view. Nodal spines are not considered.
PPW	Postpetiole width. Maximum width of postpetiole in dorsal view.
SL	Scape length. Maximum straight‐line scape length excluding the articular condyle.
SPST	Propodeal spine length. Distance between the center of propodeal spiracle and spine tip. The spiracle center refers to the midpoint defined by the outer cuticular ring but not to the center of actual spiracle opening that may be positioned eccentrically.
SPTI	Apical propodeal spine distance. The distance of propodeal spine tips in dorsal view; if spine tips are rounded or truncated, the centers of spine tips are taken as reference points.

### 2.3 Statistical analysis

All data handling and statistical data analyses were carried out in R (v. 4.0.5, R Core Team, 2021). Before analyses, we visually checked the measurements and identified four outlier specimens (AntWeb identifiers CASENT0916693, CASENT0916694, CASENT0906041, and CASENT0906013). These individuals showed substantial deviations in several traits and heavily distorted the statistical results. We excluded these specimens from subsequent analyses.

We used a modified signed‐likelihood ratio test (MSLRT) for equality of coefficients of variation to see if measurement methods (microscope versus software) yield values of different variability, separately for each trait, with the R‐package “cvequality” (Marwick & Krishnamoorthy, 2019). We utilized mixed‐effects linear regression modeling (LMM) to test whether measurement methods yield significantly different values. To fit the LMMs we used the “lme4” (Bates et al., 2015) and “lmerTest” (Kuznetsova et al., 2017) R‐packages. We fitted separate models for each trait. In each model, microscope measurement was the response, and software measurement was the predictor variable. To compensate for the fact that for each specimen, we had three repeated measurements from the microscope but only one measurement from the software. We used AntWeb ID as the random effect to control for pseudo‐replication in the response. Measurement values were re‐scaled before analyses by z‐score transformation (i.e., subtracting the arithmetic mean from all values, then dividing by standard deviation) separately for each trait. Also, in the models, software measurements were used as an offset.

From these LMMs, we could test a series of questions important for evaluating the applicability of software‐based measurements. As a preliminary step, we quantified the precision of microscope measurements based on the three repeats as the random effects variance divided by the sum of random effect and residual variance (repeatability). This was important because we treated the microscope measurements as the etalon for assessing the reliability of the software measurements. To quantify the goodness of fit between the measurement methods, we applied two approaches. First, we assessed marginal and conditional *R* ^2^ (*R* ^2^m and *R* ^2^c, respectively) for the fitted models based on the estimation method for mixed‐effects models in the *R*‐package “MuMIn” (Bartoń, 2009). Second, we estimated a standardized slope parameter (coinciding with Pearson's rho), which we acquired by re‐fitting the models with values z‐score transformed separately for measurement methods (i.e., for the given trait both microscope and software measurements had an arithmetic mean of 0, and standard deviation of 1). We were also interested in whether trait values (i.e., small or large) affected the fit between the measurement methods. We tested it by testing the null hypothesis that the regression slope is equal to 1 (i.e., there is no systematic bias in the association between measurement methods). Finally, we also assessed if there were systematic differences in measurements between the two methods (i.e., if there are significant method differences in average measurement values). Note that systematic differences might not affect goodness of fit but might have large consequences for the biological interpretations. Since trait values were re‐scaled, we could test the systematic method‐based differences by testing if the intercept of the regression slope significantly differs from zero. Significant positive or negative intercept estimates indicate that microscope measurements tend to be either larger or smaller than software measurements.

After model fitting, we used the R‐package “fdrtool” (Strimmer, 2008) to assess the value of the false discovery rate from a large number of models, for which we used the parameter estimate *p*‐values to get local false discovery rates (LFDR). We considered estimates significant if LFDR was below .05.

## 3 RESULTS

The within‐specimen agreement between repeated measures on the microscope was high in all traits, based on the estimated precision values (ranging between 0.80 and 0.97, see Table 2). This showed that the traditional approach is precise and an appropriate standard to compare the methods against.

**TABLE 2:** Model parameter estimates and Pearson's *ρ* describing the associations between microscope and software measurements of the different measured traits, as well as the estimated precision of microscope measurements based on random‐intercept and residual variance.

Trait	Number of specimens	Intercept estimate	*P* (intercept)	Slope‐bias estimate	*P* (slope)	Precision	Pearson's *ρ*	Marginal *R* ^2^	Conditional *R* ^2^
CL	42	0.187	.000	−0.044	.042	0.802	0.989	0.978	0.996
CW	29	0.282	.000	−0.025	.387	0.925	0.988	0.975	0.998
ELmax	23	0.018	.692	−0.057	.208	0.930	0.982	0.950	0.996
FRS	42	0.240	.000	0.015	.527	0.826	0.987	0.974	0.995
ML	39	−0.140	.000	0.001	.978	0.927	0.987	0.974	0.998
MW	40	0.116	.000	−0.005	.804	0.910	0.992	0.983	0.998
PEL	37	−0.068	.045	−0.031	.355	0.960	0.980	0.959	0.998
PEW	41	0.051	.070	0.034	.237	0.874	0.984	0.968	0.996
PPW	42	0.123	.000	−0.027	.210	0.849	0.989	0.978	0.997
SL	38	0.594	.000	−0.120	.040	0.970	0.932	0.864	0.996
SPST	39	0.072	.012	−0.007	.791	0.949	0.986	0.972	0.999
SPTI	42	0.151	.000	−0.008	.712	0.940	0.991	0.981	0.999

**FIGURE 2:**
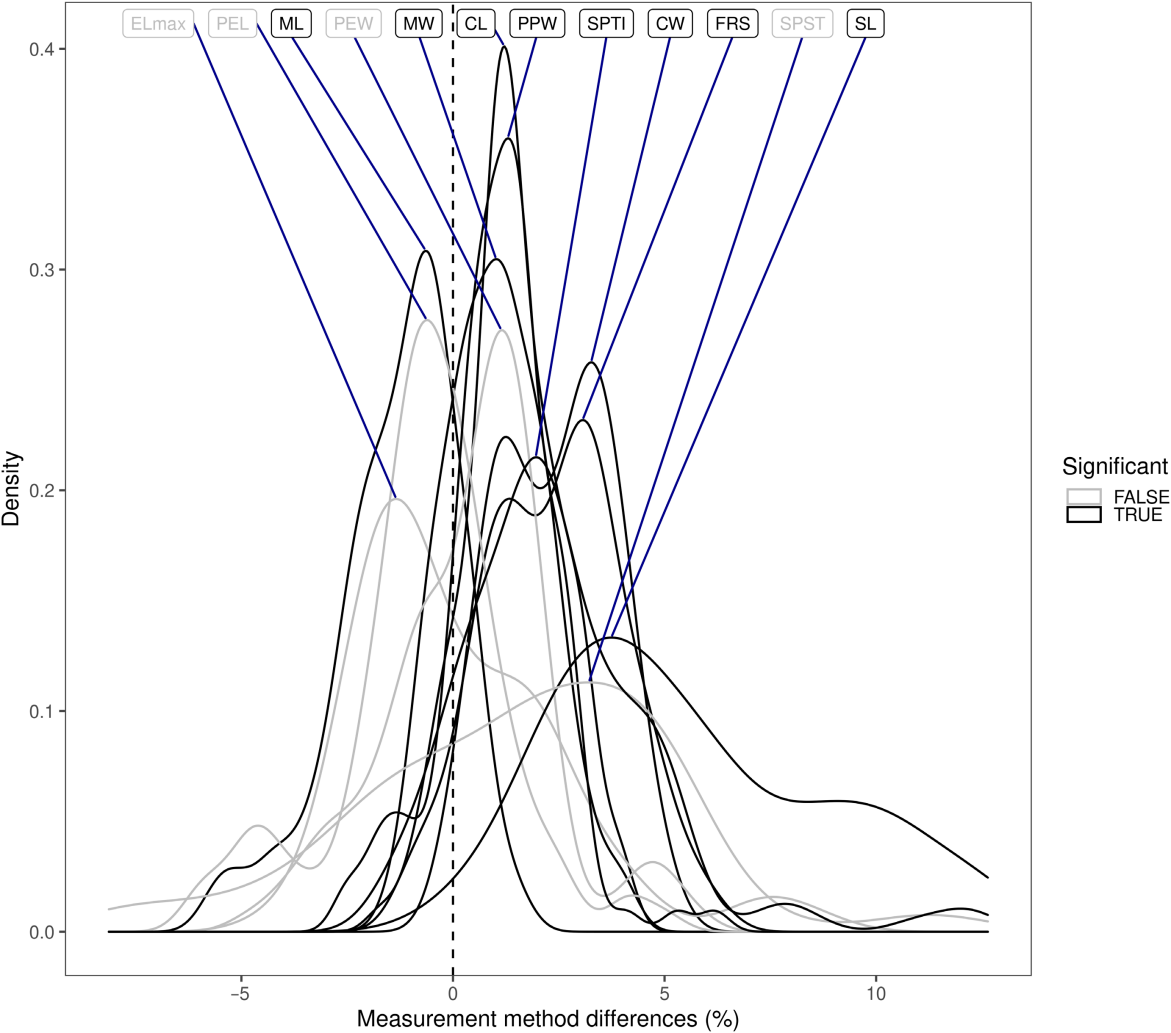
Visualization of method differences in measurement values for each trait. In each observation, the measurement method difference was calculated by subtracting the software measurement from the microscope measurement, then dividing by the microscope measurement (×100). Black and gray lines represent significant and non‐significant method differences for the given trait, respectively, assessed based on local false discovery rate (LFDR) values. Abbreviations in the figure are as follows: CL, cephalic length; CW, cephalic width; Elmax, diameter of the compound eye; FRS, frontal carina distance; ML, mesosoma length; MW, mesosoma width; PEL, petiole length; PEW, petiole width; PPW, postpetiole width; SL, scape length; SPST, propodeal spine length; SPTI, apical propodeal spine distance.

Measurement methods did not differ in their coefficients of variation for any of the traits (all *p* > .28; Table 3). The agreement between microscope and software measurements was particularly strong (as shown by *R* ^2^m [>.86], *R* ^2^c [>.99], and Pearson's *ρ* [>.93]; Table 3). We did not find evidence for a trait value effect on the agreement, that is, the slope of the regression line estimates did not significantly differ from 1 in any of the tested traits (Figure 1; Table 3). However, in eight out of the 12 tested traits, the two measurement methods showed significant differences in their mean values, as their intercept estimates were significantly different from zero (Figures 1 and 2; Table 2). Measurements from the microscope tended to be larger than those from software in seven traits, and smaller in one trait. (Figure 1 and Table 3).

**TABLE 3:** Results for the modified signed‐likelihood ratio tests (MSLRT) comparing coefficients of variation (CV) between microscope and software measurements, separately for each trait (*p* > .05 indicate no significant between‐method difference in CV).

Trait	MSLRT	*p*
CL	0.131	.717
CW	0.130	.718
ELmax	0.070	.791
FRS	−0.025	1.000
ML	0.024	.878
MW	0.073	.787
PEL	0.024	.876
PEW	0.085	.771
PPW	0.018	.894
SL	1.186	.276
SPST	0.105	.746
SPTI	0.116	.734

## 4 DISCUSSION

The most salient finding of the present study is that even though software analysis of digital images from the AntWeb repository provided data showing very high agreement with data provided by the traditional microscopic measurement method, without trait value dependence or a change in variances, there was significant systematic bias between the two methods in two‐thirds of the traits analyzed. These results draw attention to how misleading a simple analysis of between‐method agreement (e.g., statistical correlation or repeatability) can be. Herein, we summarize what can be learned from our study testing the reliability of new methods, particularly regarding the benefits and pitfalls of using AntWeb images.

### 4.1 Challenges and advances in testing inter‐method reproducibility

We advocate that more than simply testing the agreement between different approaches/methods is a necessity to evaluate true measurement reproducibility. This is because relatively high repeatability (estimated even with state of the art statistical approaches: Nakagawa & Schielzeth, 2010; Wolak et al., 2012; Wylde & Bonduriansky, 2021) alone would not ensure free interchangeability between or mixing of measurements made using different methods. Assuming significant and high repeatability, there are still a couple of other problems to be considered. For instance, variances can change. If the new method is less accurate than the traditional method, repeatability might be still considerable, but the power to detect patterns will drop. Furthermore, the direction of variance change can be trait‐specific, rendering patterns provided by the different methods hard to compare and making the pooling of data collected with different methods risky. Another problem is that the level of agreement can be trait value‐specific (i.e., stronger in higher and weaker [or even lacking] in lower values or vice versa). Negative effects stemming from this problem are hard to clearly foresee. For instance, if one wants to compare groups (taxa, sexes, populations) that are different in mean trait value, trait value‐specific agreement can be a real problem when applying a new method that is expected to be more efficient (i.e., faster, cheaper, easier, and more accessible) than the traditional one that can provide “true” values. Finally, the least expected problem can occur when agreement is high, there is no trait value dependence in agreement, and the variances remain unchanged, but there are systematic differences in the values gathered by the different approach (i.e., one method systematically produces higher/smaller values than the other). In this case, the new method provides estimates that are similar in precision to the traditional one, but yields lower accuracy. In such a case, the new method is fine for any studies where the actual values are not important because one is interested in their relative differences, so long as data from the two methods are not pooled. For instance, one could use this method to establish trends or differences between groups (e.g., sexual dimorphism, phenotypic plasticity, variation along ecological gradients), but the data themselves could not be used to describe biological phenomena (e.g., taxonomic descriptions). Our case of using digital images from the AntWeb repository for measuring morphological traits with software as a surrogate for measuring actual specimens under microscope fell in this last problem category.

### 4.2 Recognizing pitfalls in a virtual collection in morphometry: The case of AntWeb

Traditional morphometry of small invertebrates relies on measurements done under a microscope. This approach relies on expensive equipment and highly trained personnel. With proper equipment, accuracy is expected to be high since we are measuring the traits directly. Our equipment is appropriate for this purpose and has been used for ant morphometry in several studies (Csősz et al., 2015; Csősz & Fisher, 2015). However, precision is highly dependent on the person performing the measurements. In our case, based on three independent repeats, we detected high precision, so our microscopic measurements of ant linear traits are adequate to serve as an etalon for comparisons with new methods. The new method we were interested in was measuring digital images freely accessible to anyone from the AntWeb repository, using the also freely accessible and widely used tpsDig software. The increasing popularity of AntWeb among myrmecologists is easy to understand: there is no need for researchers to travel to or transport the specimen, no need for expensive microscope setups, and no need for intensive training to produce the measurements. Instead, one can download high‐resolution images and measure them with any of the available open‐access measurement software using almost any personal computer. One would intuitively assume that the two methods are identical, since ant traits cannot be measured by hand, and positioning under the microscope for photography is similar. This was perhaps the reason why no formal tests of reproducibility were made with AntWeb (or any other) digital images.

Our preliminary results were promising: the software measurements showed exceptionally high agreement with the otherwise highly precise microscopic measurements. In many cases, researchers, including authors of the present paper, would have felt satisfied that the methods were similar and stopped at this point (Csősz et al., 2021). The lack of trait value dependence in agreement and the lack of variance changes were even more promising. However, the significant systematic biases detected in eight out of 12 traits are worrying. Most body parts have been perfectly aligned in the digital images, and the bias (where detected) can be ascribed to the method's bias. However, some body parts, particularly appendages (i.e., antennae, legs), are vulnerable to alignment issues. Each trait, when measured, must be perpendicular to the axis of the optics, which can be checked using the depth of field in a stereomicroscope. A body part is perfectly aligned for measurement when both measurement points are in focus. In virtual specimens, there is no option to check alignment via depth of field and focus because these images are made up of a combination of a number of composite images, masking setup problems after all images are concatenated into 1 z‐stack image. This means that in the photo, seemingly well‐adjusted body parts (i.e., deceptively, both endpoints are in focus) are not perpendicular to the axis of the measuring optics, resulting in a false, smaller morphometric value for the given trait. This could be one explanation for the pattern of software measurements being systematically smaller than microscope measurements that we found for several traits. Furthermore, this might be the reason behind the two outlier individuals (that were omitted from the analyses) showing extreme differences in scape length (SL) and cephalic length (CL) between the perfect‐looking digital images and microscopic measurements. When we revisited these specimens, we found that our microscope measurements were correct.

### 4.3 Conclusions

Our results clearly demonstrates that introducing and establishing a new method/approach, analyzing agreement with the traditional approach via statistical correlation, goodness of fit or repeatability is not enough, and accepting a new methodology based solely on this can have serious effects on study outcomes and reliability of conclusions. Our particular case resulted in mixed conclusions. The (relatively) new methodology was the inexpensive and quick software‐based measurements of digital images from the AntWeb repository requiring minimal training, proposed as an alternative to the technique requiring expensive equipment, relatively slow microscope measurements, considerable training, and often the travel of researchers or transportation of valuable museum specimens for the morphological measurements of ants. We found exceptionally high agreement between the new and traditional methods, while we found no trait‐value dependence of the agreement or any effect of methodology on trait variance. However, we found considerable systematic bias, as software measurements typically underestimated microscope measurements. This suggests that AntWeb‐derived morphological data are useful whenever the absolute values are not important, for instance, for comparing sexes, populations/species adapted to different habitats, or treatment groups from experiments, and can even be useful for taxonomy as long as the goal is to separate groups. However, the data cannot be trusted when the actual values are of crucial importance, such as in taxonomic descriptions or in classification keys. Furthermore, we strongly advise against pooling the two types of data directly. One solution to the problem can be calibration. Using enough (>20) individuals from the model taxa with both types of measurements available, calibration for the systematic bias would be possible. At any rate, before using AntWeb data for studies where the actual values are important, or to pool the different types of data, we recommend performing a similar analysis to the one we presented here, and base the decision about the best approach on the results.

### 4.4 Prospects and a guideline on the applicability of AntWeb specimens as a source of morphometric data

When are AntWeb digital photograph measurements suitable for ant research?

1. Virtual collection constitutes the single available data source in the research. General eco‐evo studies, species descriptions, or identification keys where different groups (sexes, populations, or even different taxa) are compared for detecting differences in morphological characters. AntWeb constitutes a reliable source for such research since the goodness of fit between the two methods was exceptionally high, with no sign of differences in variation or trait‐value‐dependent patterns, and data collected from virtual specimens from this depository show high internal consistency.
2. Complex studies where data from virtual specimens and microscopic measurements are integrated. In taxonomic studies, where researchers need to integrate classic microscopic measurements with some morphometric data gathered from virtual specimens (e.g., types, or individuals from exotic or inaccessible places) inferences should be done with extreme care. In this case, analyses become vulnerable to systematic differences in trait sizes, which in turn may lead to false interpretations of findings.
  - Calibration: In some cases, a method comparison, that is, comparing the microscopic data to the measurements made from the images of the online database. Such calibration can be achieved by observing at least 20 individuals via both methods. With this procedure, most errors resulting from the difference between the methods can be eliminated. This procedure is recommended when large amounts of measurement data from both methods (i.e., microscope and software measurements from the virtual collection) are ready to be integrated.
  - Measure multiple specimens: To supplement a large number of microscope measurements with a few virtual individuals, as needed in taxonomic studies where the bulk of the data come from a microscope, but a few type specimens are available solely via a virtual collection. In such cases, the number of individuals available in the online databases is typically small. Still, several individuals can often be photographed from the collections of different museums. If more individuals are available, it is advisable to measure them all, and then check whether any outliers are among them. If so, we perform the analysis without the outlier or the poorly positioned body part, and the results should be scrutinized with care.
  - Individual screening: In some instances, calibration and multiple units are not available. In situations like this, it is worth proceeding with caution. In our research, 9% of the cases were outliers. Two specimens out of 46 (4%) had to be removed due to scale issues. Two others provided outlying morphometric values, most likely due to the wrong alignment. This underscores the problem with singletons. To work around this issue, we evaluate the data visually using a matrix scatterplot, looking for outliers of some characters. If signs of an error in any trait (outlier) are found, analyses should be conducted without the affected body part.

Finally, the above suggestions are only valid for those cases when all body parts of the specimen in the picture can be clearly measured. In a small number of cases, cursory inspection of the virtual specimen may reveal that it has suffered a major damage during preparation or storage, making morphometric observations impossible.

Our results complement the existing literature on factors that may influence the measurement results of morphometric studies (David et al., 1999; Seifert, 2002; Wylde & Bonduriansky, 2021), and may help guide the development of future online image databases. In light of this, we believe that the virtual access and examination of specimens preserved in scientific collections will facilitate research in insect morphology. However, our work highlights the importance of in‐person examination of specimens using well‐established microscopy methods.

## AUTHOR CONTRIBUTIONS

**Sandor Csosz:** Conceptualization (equal); data curation (equal); methodology (equal); writing – original draft (equal). **Ferenc Báthori:** Conceptualization (equal); data curation (equal); methodology (equal); writing – original draft (equal). **Zoltán Rádai:** Conceptualization (equal); formal analysis (equal); methodology (equal); software (equal); visualization (equal); writing – original draft (equal). **Gábor Herczeg:** Conceptualization (equal); methodology (equal); writing – original draft (equal). **Brian Fisher:** Conceptualization (equal); methodology (equal); writing – original draft (equal).

## CONFLICT OF INTEREST STATEMENT

The authors declare no competing interests.

## FUNDING INFORMATION

This study was supported by the National Research, Development, and Innovation Fund under Grant No. K 135795 (on behalf of SC), and the National Science Foundation under Grant No DEB‐1932467 Ants of the World (BLF).

## Supporting information


Table S1

## Data Availability

The data that support the findings of this study are openly available in Dryad at https://doi.org/10.5061/dryad.612jm647j.
