# CORRIGENDUM

**DOI:** 10.1002/ece3.10248

**Published:** 2023-07-03

In the recent article by Báthori et al. (2023), the authors would like to update the caption of Figure 1 as shown below:

FIGURE 1 The association between microscope and software measurements was analyzed separately for the tested traits. N is the number of specimens for which both measurement methods could be used for the given trait. p‐values represent the significance of the regression lines' slope being different from 1 (i.e., the significance of slope‐bias differences between methods, see Section 2 for details). Dashed lines denote the expected association in the case of perfect agreement between methods; solid lines represent the regression slopes from the fitted models. Abbreviations in the figure are as follows: CL, cephalic length; CW, cephalic width; Elmax, diameter of the compound eye; FRS, frontal carina distance; ML, mesosoma length; MW, mesosoma width; PEL, petiole length; PEW, petiole width; PPW, postpetiole width; SL, scape length; SPST, propodeal spine length; SPTI, apical propodeal spine distance.
